# Genomic selection signatures in autism spectrum disorder identifies cognitive genomic tradeoff and its relevance in paradoxical phenotypes of deficits versus potentialities

**DOI:** 10.1038/s41598-021-89798-w

**Published:** 2021-05-13

**Authors:** Anil Prakash, Moinak Banerjee

**Affiliations:** 1 Human Molecular Genetics Lab, Neurobiology and Genetics Division, Rajiv Gandhi Centre for Biotechnology, Thiruvananthapuram, Kerala 695014 India; 2 Department of Biotechnology, University of Kerala, Kariavattom, Thiruvananthapuram, Kerala India

**Keywords:** Developmental biology, Genetics, Psychology, Biomarkers, Diseases, Medical research, Neurology, Pathogenesis

## Abstract

Autism spectrum disorder (ASD) is a heterogeneous neurodevelopmental disorder characterized by paradoxical phenotypes of deficits as well as gain in brain function. To address this a genomic tradeoff hypothesis was tested and followed up with the biological interaction and evolutionary significance of positively selected ASD risk genes. SFARI database was used to retrieve the ASD risk genes while for population datasets 1000 genome data was used. Common risk SNPs were subjected to machine learning as well as independent tests for selection, followed by Bayesian analysis to identify the cumulative effect of selection on risk SNPs. Functional implication of these positively selected risk SNPs was assessed and subjected to ontology analysis, pertaining to their interaction and enrichment of biological and cellular functions. This was followed by comparative analysis with the ancient genomes to identify their evolutionary patterns. Our results identified significant positive selection signals in 18 ASD risk SNPs. Functional and ontology analysis indicate the role of biological and cellular processes associated with various brain functions. The core of the biological interaction network constitutes genes for cognition and learning while genes in the periphery of the network had direct or indirect impact on brain function. Ancient genome analysis identified de novo and conserved evolutionary selection clusters. The de-novo evolutionary cluster represented genes involved in cognitive function. Relative enrichment of the ASD risk SNPs from the respective evolutionary cluster or biological interaction networks may help in addressing the phenotypic diversity in ASD. This cognitive genomic tradeoff signatures impacting the biological networks can explain the paradoxical phenotypes in ASD.

## Introduction

Autism spectrum disorder (ASD) is a heterogeneous neurodevelopmental disorder characterized by impairments in communication, social interaction, and restricted or repetitive behaviors. While ASD involves reductions in verbal skills but on the positive side, it also shows increased focus of attention^1^. Overall ASD is characterized with below-average Intelligence Quotient (IQ), in contrast it is also discussed as a disorder of high intelligence^2^. Therefore, on one side it is a result of deficits in brain function resulting in impaired social behavior, communication and language, while on the other side it also demonstrates gain in brain function as evident from increased auditory pitch perception, increased visual-spatial abilities, enhanced synaptic functions^3–8^. Some of these gain in brain function might influence the capability of ASD individuals towards increased attention to detail, better observation skills, focused concentration, ability to absorb and retain facts, (a feature often associated with long term memory), better visual imaginative skills (where they think in pictures), greater analytical skills (as they can spot patterns and repetitions which are common in subjects such as Science, math and music), unique and creative thought processes resulting in innovative solutions, increased tenacity and resilience^9^. Evolutionarily, in comparison to apes, the human brain size has tripled, that impacted brain organization and functions^2^. Contrastingly increased brain size, rapid brain growth or increased synaptic functions can impact brain function in either way depending on where the growth is and how the synapses interact^10–13^. How common or rare are these deficits or gain in function in ASD is not well understood. But possibly this would largely depend on their genomic makeup and early developmental environment that nurtures this gain in functions. It has been demonstrated that various phenotypic variables that are a part of ASD such as learning, communication, speech, cognition, behavior, neurodevelopment etc. are largely influenced by its genes^14^. These phenotypic variables are known to be polygenic in nature with multiple alleles with small effect size, which may aggravate or decline depending on the nurturing environment^15,16^. Therefore, one would wonder can these paradoxical phenotypes of deficits and gain in brain function be explained by genomic tradeoff, either at genomic level or genotype phenotype level. Do these tradeoff signature has any evolutionary significance.

Ideally a Genomic Trade-off hypothesis states that certain genomic changes may tend to produce disease in a subset of individuals but are still retained in the population as they turn out to be beneficial overall. Genomic trade-offs can influence specific phenotype and human adaptations^17^. It would be interesting to identify which genes, or cluster of genes or network of genes underwent positive selection during the course of evolution and how they interact among each other. To address this query, we searched for the positive selection in all the common ASD risk single nucleotide polymorphisms (SNPs). Then went on to search for pattern of clustering or interaction of these positively selected risk SNPs, and how they reflect a biological or cellular phenotype. Do these functionally relevant positively selected risk alleles signify a genomic tradeoff and if so does it reflect a tradeoff between phenotypic traits. What is the evolutionary significance of these positively selected ASD risk alleles? Do these evolutionary domains also reflect a functionally impact on phenotypic traits as a part of human evolution?

## Results

### Identifying selection in ASD risk SNPs

We retrieved 1019 SNPs associated with ASD risk from SFARI Human gene database which includes both rare and common variants (Supplementary Table S1). From these only 446 common SNPs were having risk allele information and ethnicity data, and these were selected for further analysis. These SNPs were extracted from Phase 1 and Phase III data of 1000 genome and were subjected to selection tests. Using machine learning based method for Phase I data, only nine significant positive selections were detected out of 1338 selection tests (Supplementary Table S2). Using individual tests for positive selection, such as Fst, Tajima’s D, DAF, XP-EHH, XP-CLR that summed up to 12,042 selection tests, we identified 185 significant positive signals (Supplementary Table S3).

While testing for positive selection in Phase III data using PopHuman Genomics Browser we identified 299 positive tests from 12,042 selection tests (Supplementary Table S4). These 299 positive signals from Phase III data not only covers the positive signals from Phase I data but also adds few new selection signals. These selection signals in the ASD risk SNPs were further verified in presence of positive and negative control. As expected, all positive controls did display positive selection using all approaches. While in negative controls machine learning approaches did not identify any major positive signals but individual tests did identify few positive selection signals in randomly identified negative controls.

### Identifying global and individual level selection at ASD risk SNPs

**Figure 1:**
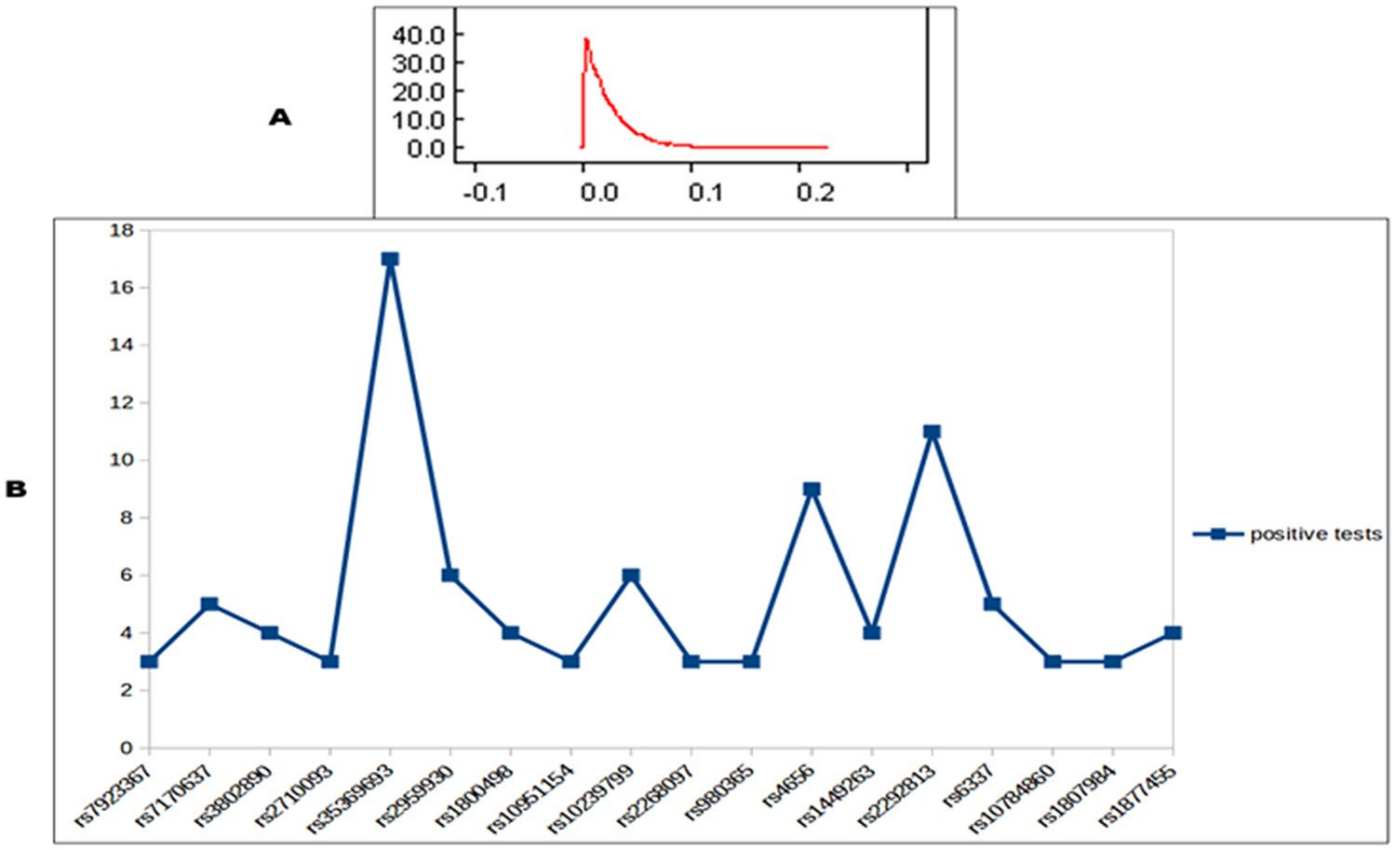
Bayesian conjugate beta-binomial analysis. (**A**) Posterior distribution obtained after 10,000 MCMC simulations. (**B**) Positive selection tests that crossed the minimum threshold.

In order to identify maximum selection at individual SNPs we performed a Bayesian conjugate beta-binomial analysis as per the criteria mentioned in the methods. Minimum one-tailed upper confidence limit was three positive tests, derived from Bayesian conjugate beta-binomial analysis (Fig. 1A). Using this stringency, we identified 61 SNPs out of the 446 SNPs that surpassed this threshold limit (Supplementary Fig. S1, Supplementary Table S5). All the positive control SNPs also passed this threshold. SNPs in which association and selections were reported in the same population and those having the same risk and the selected allele, were retrieved from these 61 SNPs. Thus only 18 SNPs were obtained and used for further functional, interaction and evolutionary analysis (Fig. 1B).

### In silico functional assessment of the selected SNPs

Majority of the positively selected SNPs were identified to have a regulatory role as evident from their Regulome DB rank (Supplementary Table S6). The missense SNP was identified to have potentially damaging role as evident from its Polyphen score. Gene expression analysis of these positively selected SNPs were extracted from GTEX portal. Majority of the SNPs do impact gene and tissue specific expression alterations and are also found to impact the brain tissues (Supplementary Table S7). Based on these observations we do suggest that these positively selected SNPs can play a significant role in altered gene expression.

**Figure 2:**
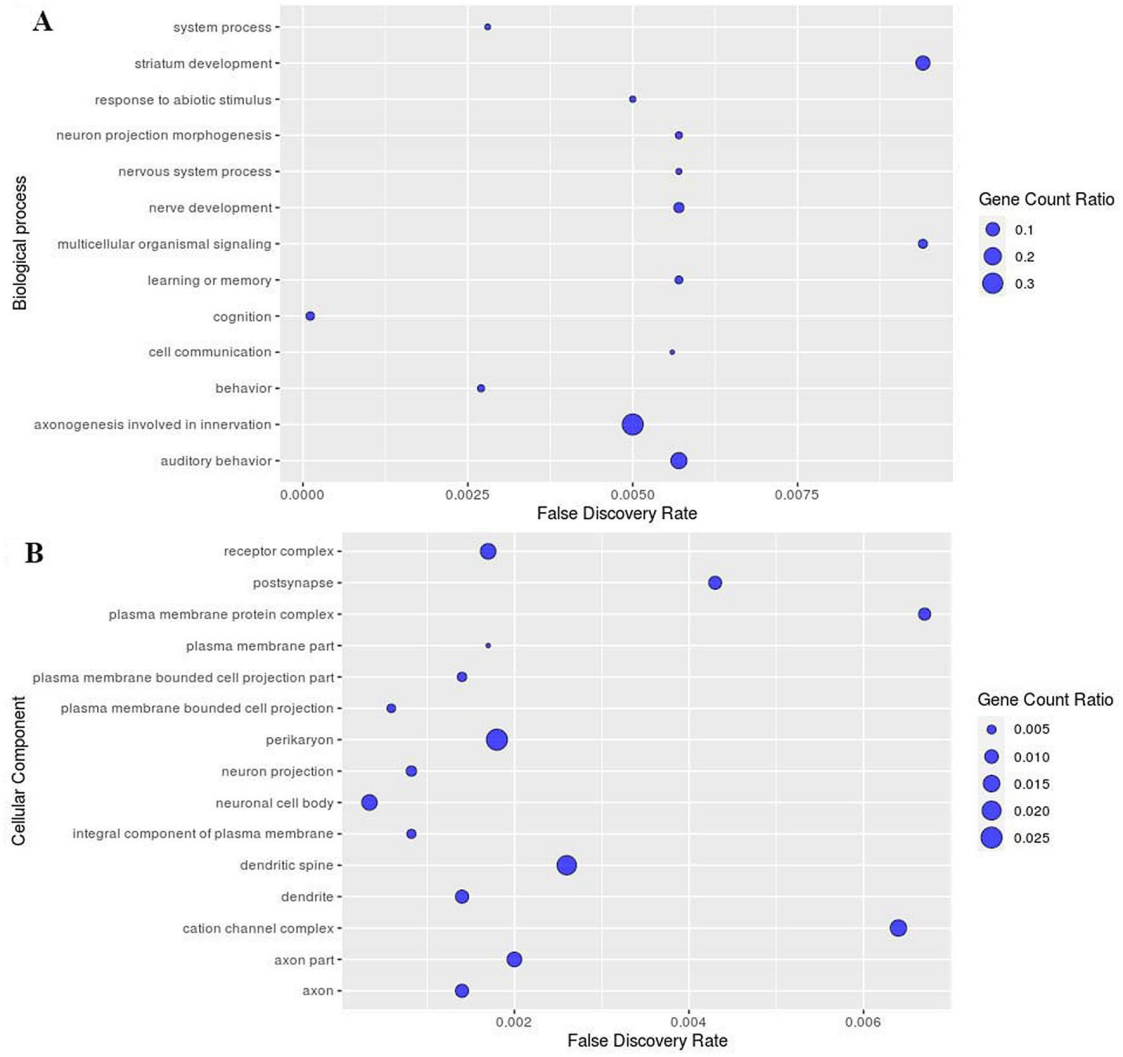
Gene ontology enrichment plots for positively selected SNPs showing (**A**) biological processes with their FDR cut off and gene count ratio (**B**) cellular processes with their FDR cut off and gene count ratio.

**Figure 3:**
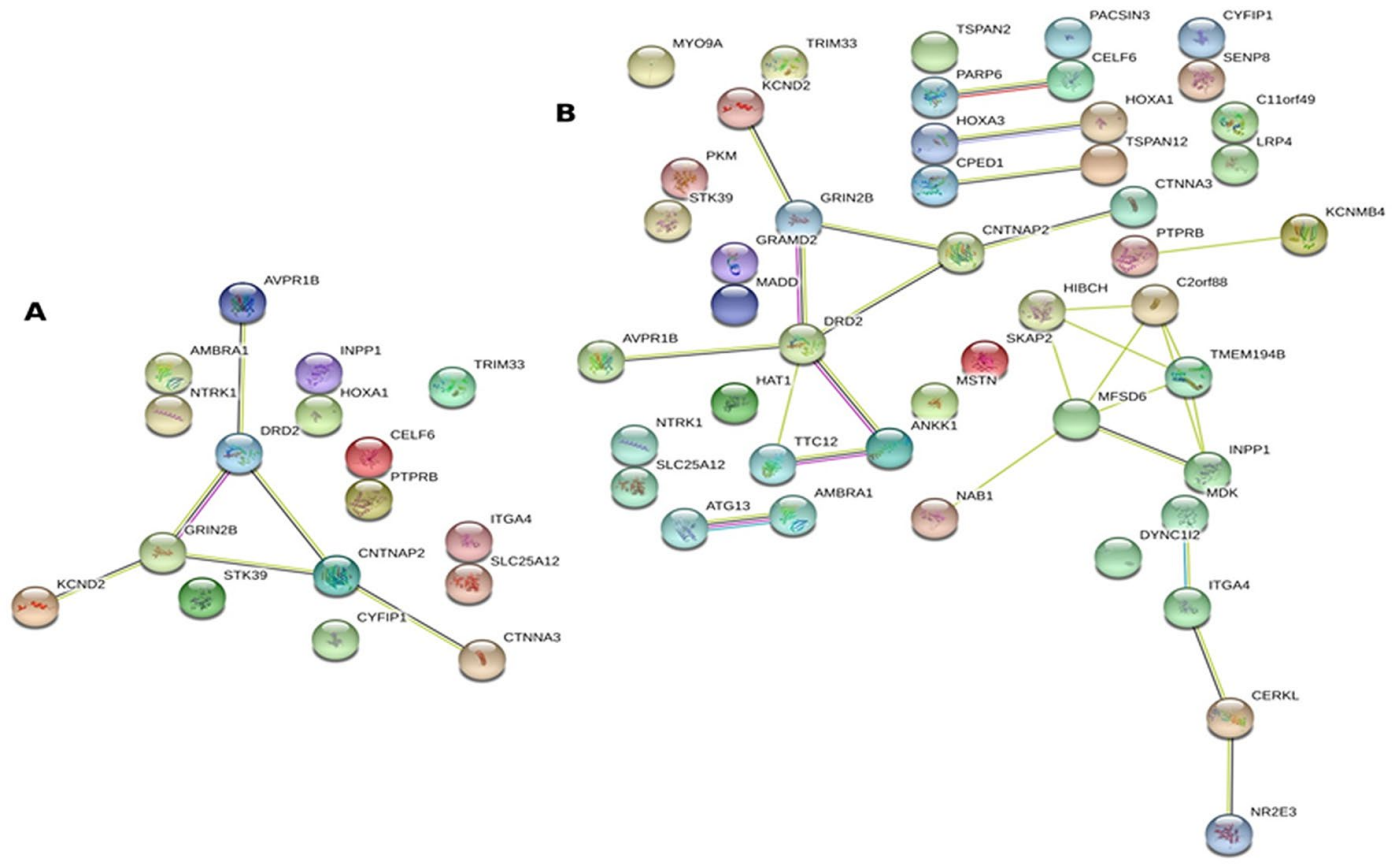
Protein–protein interaction networks. (**A**) STRING network showing genes harboring the positively selected SNPs (nearby genes for intergenic SNPs). (**B**) STRING network after including eQTL genes in the input list.

Subsequently we were keen to identify the biological and cellular processes associated with these positively selected ASD risk SNPs and their eQTL genes. Gene Ontology enrichment analysis plots with low FDR cut-off (< 0.01) predicted that several of these genes are involved in multiple biological and cellular processes associated with brain function (Supplementary Table S8). Several of these genes show enrichment for biological processes associated with cognition, behavior, system process, response to abiotic stimulus, cell communication, learning or memory, nervous system process and multicellular organismal signaling (Fig. 2A, Supplementary Fig. 2A). Various cellular components that are enriched in the Gene Ontology enrichment analysis include neuronal cell body, neuron projection, axon, dendrite, perikaryon, postsynapse, dendritic spine, cation channel complex, components of plasma membrane, and plasma membrane protein complex (Fig. 2B, Supplementary Fig. 2B). Interestingly, biological interaction network using STRING analysis show that some genes strongly interact among each other and form the core of the network, while others lie in the periphery with or without interacting with the core network. The overall Protein–protein interaction (PPI) enrichment score is statistically significant *P* = 0.038 indicating strong interaction. The genes that form the core of the network include *AVPR1B, DRD2, GRIN2B, CNTNAP2, KCND2* and *CTNNA3,* and these genes are also associated with cognition, learning and other higher order brain functions (Fig. 3A). A similar interaction network was observed with eQTL genes too but involved addition of *TTC12* and *ANKK1* joining the core with *DRD2* (Fig. 3B) to form a part of the NTAD gene cluster (*NCAM1-TTC12-ANKK1-DRD2*). The PPI enrichment score was statistically highly significant *P* = 5.01 × 10^–7^ indicating strong interaction. The genes that did not form the core of the network interacted directly or indirectly influenced the cellular and biological processes through peripheral network as evident with the interaction of *INPP1, ITGA4, SLC25A12* and *STK39* (Supplementary Tables S7, S8).

### Evolutionary history of risk SNPs

**Figure 4:**
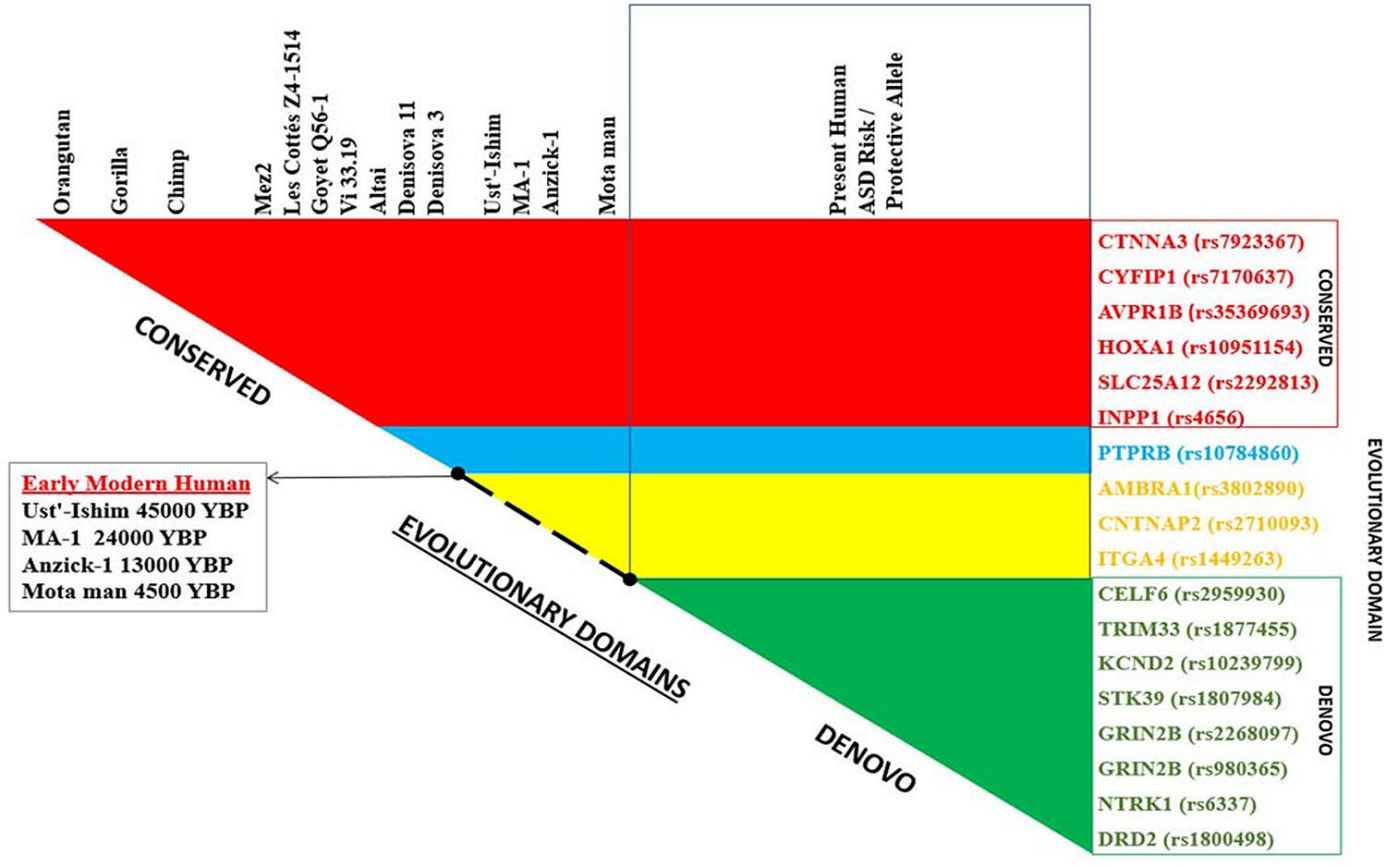
Evolutionary pattern of positively selected ASD risk loci, showing conserved evolutionary selection domain (Red), *Denovo* evolutionary selection domain (Green), Intermediate selection domain (early to recent Modern human—yellow).

The evolutionary origin of these 18 positively selected ASD risk alleles identified two evolutionary domains (Fig. 4, Supplementary Table S9). Interestingly, the risk alleles of rs1800498(A)*DRD2*, rs2268097(G)*GRIN2B*, rs980365(C)*GRIN2B*, rs6337(T)*NTRK1*, rs1807984(G)*STK39*, rs10239799(C)*KCND2* and protective alleles of rs1877455(T)*TRIM33*, rs2959930(G)*CELF6* are present only in recent modern humans. This allelic selection of positively selected ASD risk SNPs of *DRD2*, *GRIN2B*, *GRIN2B*, *NTRK1*, *STK39*, *KCND2* and protective alleles of *TRIM33*, *CELF6* are referred as *Denovo* Evolutionary Selection Domain as it was not observed in any of the ancestral species, including early modern humans. This *Denovo* Evolutionary Selection Domain that mostly comprises of genes pertaining to cognition and learning seems to have evolved in the last 4500 years, as evident from the variant sites that were found to be missing in the Motaman, that dates back to 4500YBP and even Anzick1 which dates back to 13,000YBP. The risk alleles of rs3802890(A) *AMBRA1*, rs1449263(T) *ITGA4*, rs2710093(C) *CNTNAP2* were seen only in recent and early modern human suggesting to have evolved in last 45,000 years. In contrast to *Denovo* Evolutionary Selection Domain, there were certain risk alleles in ASD risk genes, rs7923367(G) *CTNNA3*, rs35369693(G) *AVPR1B*, rs2292813(C) *SLC25A12*, and rs10951154(T) *HOXA1* that were found to be conserved throughout the evolutionary time scale, starting from primates to modern humans. This evolutionary selection domain is referred as Conserved Evolutionary Selection Domain. However, few exceptions with interrupted evolution such as rs4656 (G) *INPP1* risk allele and the protective allele of rs7170637(A) *CYFIP1* were also found to be conserved throughout the evolutionary time scale but with contrasting interruptions. While rs4656(G) *INPP1* risk allele was not seen in Neanderthals and Denisovans but reemerged in early modern humans in contrast the protective allele of rs7170637(A) *CYFIP1* was present in primates to Neanderthals and reemerged in modern humans while absent in early modern humans. The protective allele of rs10784860(T) *PTPRB* is conserved in all hominin species with exception to Motaman and Denisova3.

## Discussion

The present study is one of the most exhaustive evaluation of positive selection in ASD risk SNPs and their involvement in biological, cellular and functional implication. In addition, it also predicts its evolutionary significance and implication in ASD phenotypes. Earlier studies have just reported positive selection in ASD loci, but was limited to GWAS data of Psychiatric Genomics Consortium and restricted to using machine learning tool^18^. Whereas, the present study extensively utilizes machine learning methods, different individual tests for selections using data from Phase I and Phase III and also Bayesian methods to identify positive selection in ASD risk SNPs. The study identifies a pattern of selection in ASD risk SNPs that associate with differential implication to brain functions, which indicate a cognitive genomic trade-off for ASD phenotypes.

The in silico functional evaluation of the positively selected ASD risk SNPs, do reflect a regulatory role and likely pathogenic as evident from the RegulomeDB score, SIFT and PolyPhen score. Gene ontology enrichment analysis for the ASD risk genes and their eQTL genes indicate the involvement of biological processes associated with cognition, memory, learning, behavior, neuronal development etc., while the cellular processes also support the roles of neurons, axons, dendritic spines etc. All these observations clearly indicate that the positively selected ASD risk SNP do play a significant role in impacting the higher order brain function such as cognition. Interestingly, the genes that support these higher order brain functions also form the core hub of the biological interaction network. This is evident with the involvement *AVPR1B, DRD2, GRIN2B, CNTNAP2, KCND2* and *CTNNA3* and the NTAD gene cluster that can jointly impact cognition, behavior, learning, memory and other nervous system processes associated with higher order brain function. NTAD cluster genes are known to be co-regulated and involved in nervous system development and neurotransmission^19^. These biological and cellular functions are known to be altered and their differential presentation in ASD can result in diametrically opposite phenotypes. Thus in ASD phenotypes, cognitive genomic trade-offs seems to be a plausible outcome. Evolutionary assessment of the risk SNP genes that form the core of the interaction network, indicate that they belong to the *Denovo* Evolutionary Selection Domain, while the genes in the periphery of the network belong to the intermediate or Conserved Evolutionary Selection Domains. Considering the time scale of early modern humans to recent modern humans used in the study, one can predict that this *Denovo* Evolutionary Selection Domain might have emerged within the last 4000 years. Thus the evolutionary pattern of these genomic tradeoff signature genes imply that ASD might been a casualty of higher order brain function. The phenotypic variation in gain or loss in cognitive function might also be explained by this cognitive genomic tradeoff for ASD risk SNPs, depending on the combination of risk SNPs or environmental variables.

Cognition has been one of the most prominent domains of human brain function which is unique from its other hominin species. A possible explanation to trade off hypothesis between health and disease (ASD), can be explained by possible mismatch of Evolutionary selection domains (*Denovo* and Conserved Evolutionary Selection Domains) or mismatch between the epistatic interaction among the core and peripheral network or disadvantageous combinations of allelic preferences either directly or a indirectly, through environmental insults. How epistatic or epigenetic interactions influence ASD phenotype has not been thoroughly investigated. However, limited studies on epistatic interaction between genes in the RAS/MAPK pathway in ASD have been demonstrated^20^. Similar epistatic interaction can be expected in these positively selected ASD risk loci, but needs precise investigation on how they impact phenotype variation in ASD. The genes in the peripheral network or the Conserved Evolutionary Selection Domains such as *HOXA1* and *CYFIP1* have been shown to have increased expression, resulting in ASD phenotype^21,22^. *CYFIP1* is reported to coordinate mRNA translation at dendrites^23^. Epigenetic studies on ASD risk loci are also very limited although epimutations and DNA methylations have been reported in ASD^24–26^. Altered methylations have also been reported in these core network genes such as *DRD2*, *GRIN2B* which are also likely to impact dendritic spine density, altered synaptic function, disruption of the glutamatergic/GABAergic balance^27,28^. These cellular functions are known to be altered in ASD. It has been demonstrated that *DRD2* methylation can alter cognitive function and reduced prefrontal dopaminergic activity has also been reported in ASD phenotype^29,30^. Interestingly, several genetic variants and *denovo* mutations in the genes that influence DNA methylations such as *DNMT3A, TET2, MECP2, MBD5* have been reported to be associated with ASD^31,32^. These observations might clearly indicate a possible role of epigenetic modifying enzymes, resulting in epigenetic dysfunction. A complex interplay of genetic networks and allelic selection of genes involved in cognition might have been critical in developing higher order thinking processes in humans. Allelic imbalances in these genetic networks might also drive the human species into a functional state of the brain which may not seem to be normal. Therefore, determining the epistatic or epigenetic interactions may demonstrate the direction of the function, whether gain or loss of function in ASD phenotype. A precise understanding of this cognitive trade-off might therefore, help in understanding the phenotypic variations in behavior spectrum of ASD patients.

Evolutionary benefit of genetic variants due to selection advantage resulted in evolution of the human brain. But in a few individuals these resulted in cognitive disorders^33^. The *Denovo* Evolutionary Selection Domain, while on one side reflects the positive side of human evolution, more importantly cognition; contrastingly it also reflects its involvement with ASD. Similarly, ASD is also characterized by impaired social skills, communication problems, and repetitive behaviors and contrastingly, certain cognitive abilities such as music, mathematics, or memory are greatly enhanced in ASD individuals and can greatly surpass the overall level of functioning of modern humans^2,34,35^. These traits are more associated with enhanced analytical capabilities. These enhanced analytical capabilities might be linked with increased dendritic spine density, activity and synaptic plasticity^36,37^ which are reported to be altered in ASD^3,34,35^. Interestingly, these traits are also associated with the genetic variants that imply the role of *Denovo* Evolutionary Selection Domain. Common genetic risk variants for ASD were reported to be positively associated with general cognitive ability, vocabulary, verbal fluency and logical memory^38^. It has been reported that highly duplicated Olduvai sequences are beneficial in cognitive development, but differences in gene dosage can result in either ASD or Schizophrenia^33,39^. Many of these cognitive functions that are associated with ASD are also likely to be influenced by educational attainment^40^. Increased educational attainments have been linked to enhanced cognitive skills in ASD. This increased educational attainment reflects either training of genes to their maximal potential or through epigenetic modification thus reflecting that *Denovo* Evolutionary Selection Domain has the potential to undergo modification. Repetitive behavior is also one of the prominent features of ASD and this feature is also evident in primates^41,42^. *SLC25A12* has been reported to be associated with restricted repetitive behavior traits^43^ and interestingly the risk variant is also conserved throughout the evolution indicating its support to conserved evolutionary domain of brain function. A precise understanding of genetic variants in different evolutionary selection domains and their relationship with various phenotypes might provide deeper insights into the phenotypic variation in ASD. Determining the enrichment of the evolutionary selection domain might also indicate how evolution of higher order brain function turned out to be a casualty resulting in ASD.

Genomic trade-offs signature in ASD indicate cognitive genomic trade-offs, reflecting on either gain or deficits in brain function. This cognitive genomic trade-off seems to be a plausible outcome of human evolution which is dominated by the *denovo* evolutionary selection domain. *Denovo* Evolutionary Selection Domain might have emerged within the last 4000 years. The trade-off between health and disease and phenotype will depend on the ordered or disordered combination of genes, either through epistatic or epigenetic interaction within or between the biological networks (core/peripheral), or within or between the evolutionary selection domains (*denovo*/conserved). Identifying the enrichment of the SNPs in the biological network or the evolutionary selection domain can provide critical clues on the ASD phenotype diversity. Since ASD is characterized by both deficits and gain in brain function, therefore, understanding the pattern of cognitive genomic tradeoff signature may explain the paradoxical phenotypes in ASD. Enrichment of genomic variants associated with enhanced cognitive function or core biological network or *denovo* evolutionary selection domain, can result in gain in brain function. In contrast when the enrichment of the risk SNPs of the genes of peripheral biological network or in the conserved evolutionary selection domain, may reflect on deficits in brain function associated with impaired social behavior, communication and language.

## Methods

**Figure 5:**
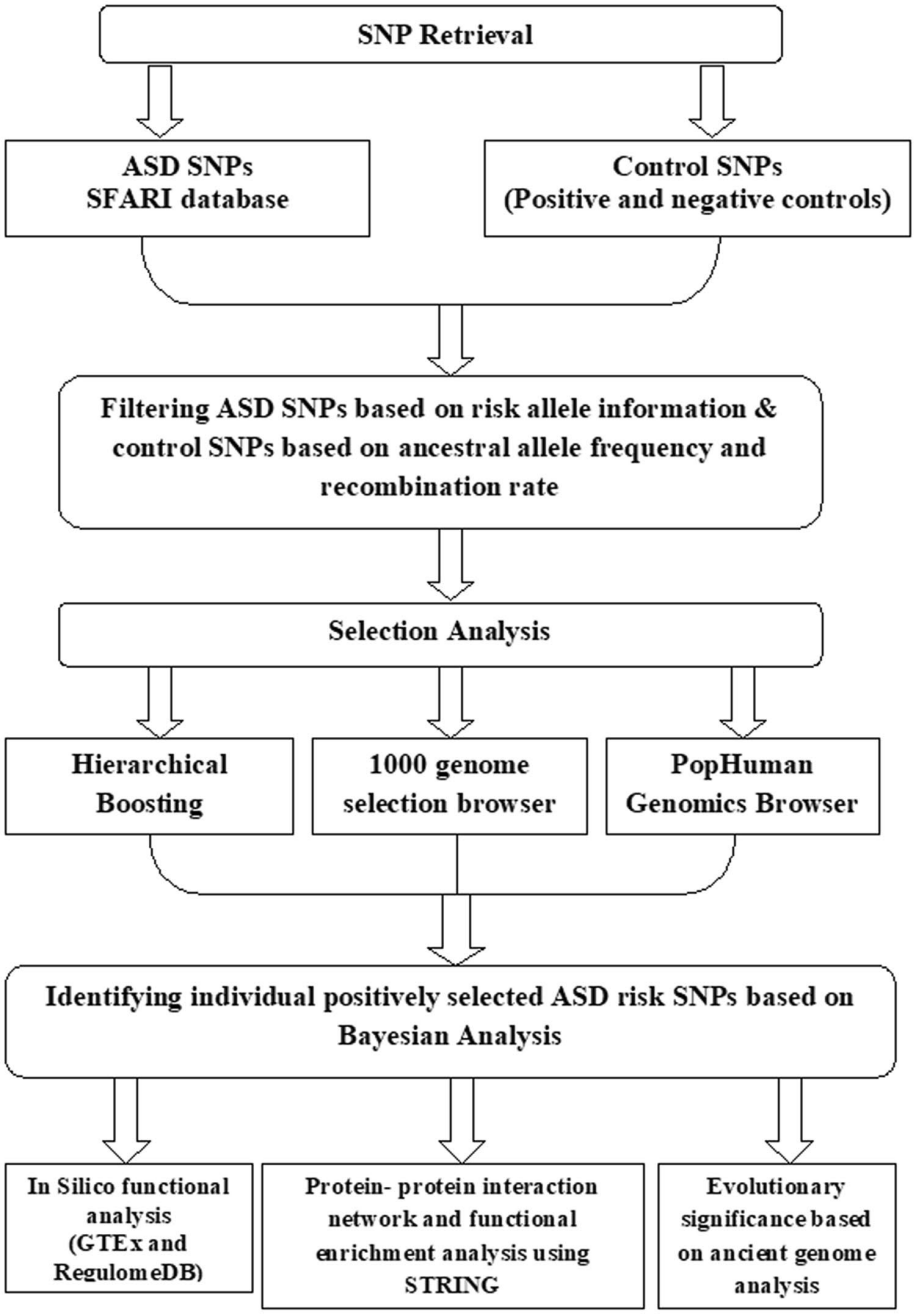
Flowchart of step-wise methodology followed in the present study.

To investigate the positive selection in ASD associated genes, SFARI database was used for mining the ASD risk genes and checked for common variants^31^. SFARI dataset were defined for ASD as per the diagnostic tools and exclusion and inclusion criteria elaborated in the link (sfari.org/ssc-instruments) Subsequently, various selection tests using individual and global approaches were used to identify whether these common risk variants are positively selected in the general population. The entire methodology is presented in a flowchart (Fig. 5).

### Data mining of ASD related genes

Complete gene lists of 1019 SNPs that were reported to be associated with ASD were retrieved from the SFARI Human gene database (gene.sfari.org/database/human-gene/). As per the SFARI dataset classification only the common SNPs were filtered and variant type and identification of the SNPs were determined using Ensembl (www.ensembl.org/index.html). Ethnicities of samples used in each study and risk allele status of each SNPs were identified by manual inspection of the respective publications. Therefore, based on the selection criteria of common SNPs and ethnicity of the risk allele, 446 SNPs were selected for further analysis (Supplementary Table S1).

### Selection tests

For the curated ASD risk SNP various selection tests were performed in Phase I and III data of 1000 genome database. For the Phase 1 data (www.internationalgenome.org/category/phase-1/), 1000 Genomes selection browser 1.0 available at hsb.upf.edu/ was used^44^. Analysis was carried out in three Metapopulations: CEU (Utah residents with Northern and Western European ancestry from the CEPH collection), YRI (Yoruba in Ibadan, Nigeria) and CHB (Han Chinese in Beijing, China) using a ‘Hierarchical Boosting’ machine-learning algorithm that combines multiple tests to give an overall view of selection. Hierarchical Boosting method implemented in 1000 genome selection browser uses a supervised boosting algorithm for classifying genomic regions based on positive selection^45^. Summary statistics of individual selection tests are used as input variables for the boosting regression functions. Some selection tests which are correlated and unsuitable for the framework are removed to avoid over-fitting. Each algorithm was trained 1000 times with a 90% re-sampling of input data and the positive selection scores are validated by comparing with empirical genome-wide data. The above stated positive selection dataset was used for determining the selection signature of ASD risk and control SNPs. In addition, various individual tests for selection implemented in 1000 Genomes selection browser 1.0 were also performed using Fixation index (Wright’s FST)^46^, Tajima’s D^47^, difference of derived allele frequency (DDAF)^48^, cross-population extended haplotype homozygosity (XPEHH)^49^, cross-population composite likelihood ratio (XPCLR)^50^ and integrated haplotype score (iHS)^51^ (window size varies according to the test). For all the selection tests for Phase I data, positive selection signals were considered significant at a 1% false discovery rate (FDR) with a ranking score.

For 1000 genome phase III data (www.internationalgenome.org/category/phase-3/) the selection test was carried out using PopHuman genomics browser^52^ available at pophuman.uab.cat/. Fst and XPEHH (10 kb window size) were carried out in the same three Metapopulations: CEU, CHB and YRI. While iHS (10 kb) was carried out in several sub-populations excluding admixed American populations^53^. For all the selection tests for Phase III data, positive selection signals were considered significant at a 1% false discovery rate (FDR) and the significance threshold was set at ± 2 SD from the genome-wide mean.

### Selection of positive and negative control SNPs

To evaluate the efficiency, correctness and significance of our selection tests we used established Positive controls and some random negative controls^54^. The positive control SNPs were selected from genes already reported to be under positive selection in various populations compiled in 1000 Genomes selection browser 1.0. From here nine such SNPs were considered as positive controls. Similarly for negative controls 446 SNPs were selected based on similar ancestral allele frequency, recombination rate, and which has not been reported to be associated with ASD. Ancestral allele frequency of the SNPs in 1000 genome phase 3 sub-populations were retrieved using Ensembl REST API^55^ followed by arcsine transformation and two-sample t tests. The selection tests were repeated for the positive and negative controls and Chi-square test was done using frequency of positive tests in control and ASD SNPs.

### Determining the threshold of positive selection at individual SNPs

We further tested the threshold of selection at each individual SNPs, for this we considered all the selection tests that demonstrated selection at individual SNPs. Bayesian conjugate beta-binomial analysis was carried out using WinBUGS program^56^ to determine the threshold value for positive selection for each individual ASD risk SNPs. The parameters of the prior distribution were decided using negative control data (‘a’ = 1 to 3.8, ‘b’ varies according to mean probability of success = 0.0227 and n = 57 for binomial likelihood function). Markov Chain Monte Carlo simulations were carried out for each posterior distribution. Minimum one-tailed upper confidence limit was selected as threshold for positive selection in ASD risk SNPs.

Next, we wanted to identify the direction of selection for ASD risk SNPs. As the positive selection can occur either in the risk or protective alleles. To resolve this, we considered all the positively selected risk alleles and verified all those SNPs in which association and selection were reported in the same population using statistical tests data and allele frequency. For associations reported in mixed ethnicities, the ethnicity contributing majorly in the sample was considered, and for subpopulations absent in 1000 genome data, metapopulations with similar ethnicity and allele frequency were considered.

### Functional implication of the positively selected SNPs

To find the functional implications of the positively selected ASD risk SNPs, we performed a comprehensive analysis of the functional impact of these genes using publicly available computational prediction tools such as RegulomeDB rank (regulomedb.org)^57^. The missense SNPs were further assessed for their functional and pathological role using sequence homology-based tool (SIFT) (SIFT-sift-dna.org)^58^ and a structural homology-based method (PolyPhen-2) (PolyPhen-2-genetics.bwh.harvard.edu/pph2/)^59^. Functional significance of these SNPs was further assessed for their expression profile based on eQTL data retrieved from GTEx portal V8 (gtexportal.org)^60^. The change in expression of the eQTL genes for the positively selected risk SNPs were noted in different tissue types.

### Interaction networks

Genes belonging to positively selected SNPs present in the intronic, exonic or UTR region or in the intergenic region of a nearby gene or their eQTL genes, were subjected to STRING analysis (string-db.org/) to identify their direct (physical) or indirect (functional) interactions^61^. The STRING database interaction records are extracted from KEGG, Reactome, BioCyc, Gene Ontology and BioCarta and restricted our search for human interactions only. STRING combines probability scores from seven independent evidence channels to obtain protein–protein interaction score. This includes three genomic context (neighborhood, fusion, gene co-occurrence) prediction channels and one each for co-expression, text-mining, biochemical/genetic data and previously curated databases. Protein–protein interaction network is constructed from interaction scores above medium confidence threshold (0.4). In addition to protein interactions, STRING v11 also provides Gene Ontology enrichment analysis using classification systems implemented in Gene Ontology and KEGG, to understand the biological processes, cellular components and molecular functions involved. Functional enrichment of the positively selected SNP and their corresponding genes or eQTL genes in various biological and cellular processes are plotted using the ggplot2 package in R^62^. For each biological and cellular function, the proportion of genes with FDRs less than 0.01 for the corresponding genes and less than 0.05 for eQTL genes was calculated, which was used to evaluate the strength of the associations.

### Ancient genome analysis

To understand the evolutionary trajectory of these positively selected risk SNPs we extracted data from 21 ancestral genomes consisting of 14 ancient hominins belonging to Denisovans, Neanderthals and four early modern humans dating 2000–45,000 YBP and three primate genomes. Ancient genomes consisted of Neanderthal genomes such as Altai Neanderthal^63^, Vindija Neanderthal genomes: Vi33.16, Vi33.25, Vi33.26^64^, and Vi 33.19^65^, additional Neanderthal genomes: Feld1, Mez1, Sid1253^63^, late Neanderthal genomes: Goyet Q56-1, Les Cottes Z4-1514, Mezmaiskaya2 and Spy 94a^66^, Denisovan genome^67^ and a Neanderthal-denisovan hybrid named Denisova11^68^. These genomes span over 750,000 to 55,000 years before present (YBP). The early modern humans considered for the study span around 45,000 to 2000 YBP. Early modern human genomes were Ust'-Ishim, Europe (45,000)^69^, Oase1, Europe (35,000)^70^, MA-1, Europe (24,000)^71^, Anzick1, USA (13,000)^72^, Motaman Africa (4500)^73^, and VN41, Asia (2000)^74^. Early modern human genomes were selected from different geographical regions to represent different ethnicities during those times. Denisovan genome and other low coverage Neanderthal genomes (Vi33.16, Vi33.25, Vi33.26, Feld1, Mez1 and Sid1253) were available as tracks in UCSC genome browser (hg19) (genome.ucsc.edu/Neandertal/). For others, BAM files were downloaded and analysed using GATK4 (gatk.broadinstitute.org)^75^ and visualized using Integrative Genomics Viewer (igv.org). For Chimpanzee, Gorilla and Orangutan genomes, Cons 46-way track from UCSC genome browser (genome.ucsc.edu) was used. Data is presented wherever available for all these with the most common/ancestral SNP.

## Supplementary information

Supplementary Information 1.

Supplementary Information 2.

Supplementary Information 3.

Supplementary Information 4.
