# Supplementary figures and images for: Genomic selection signatures in autism spectrum disorder identifies cognitive genomic tradeoff and its relevance in paradoxical phenotypes of deficits versus potentialities
Source: Sci Rep. 2021 May 13;11:10245. doi: 10.1038/s41598-021-89798-w (PMC8119484; doi:10.1038/s41598-021-89798-w)

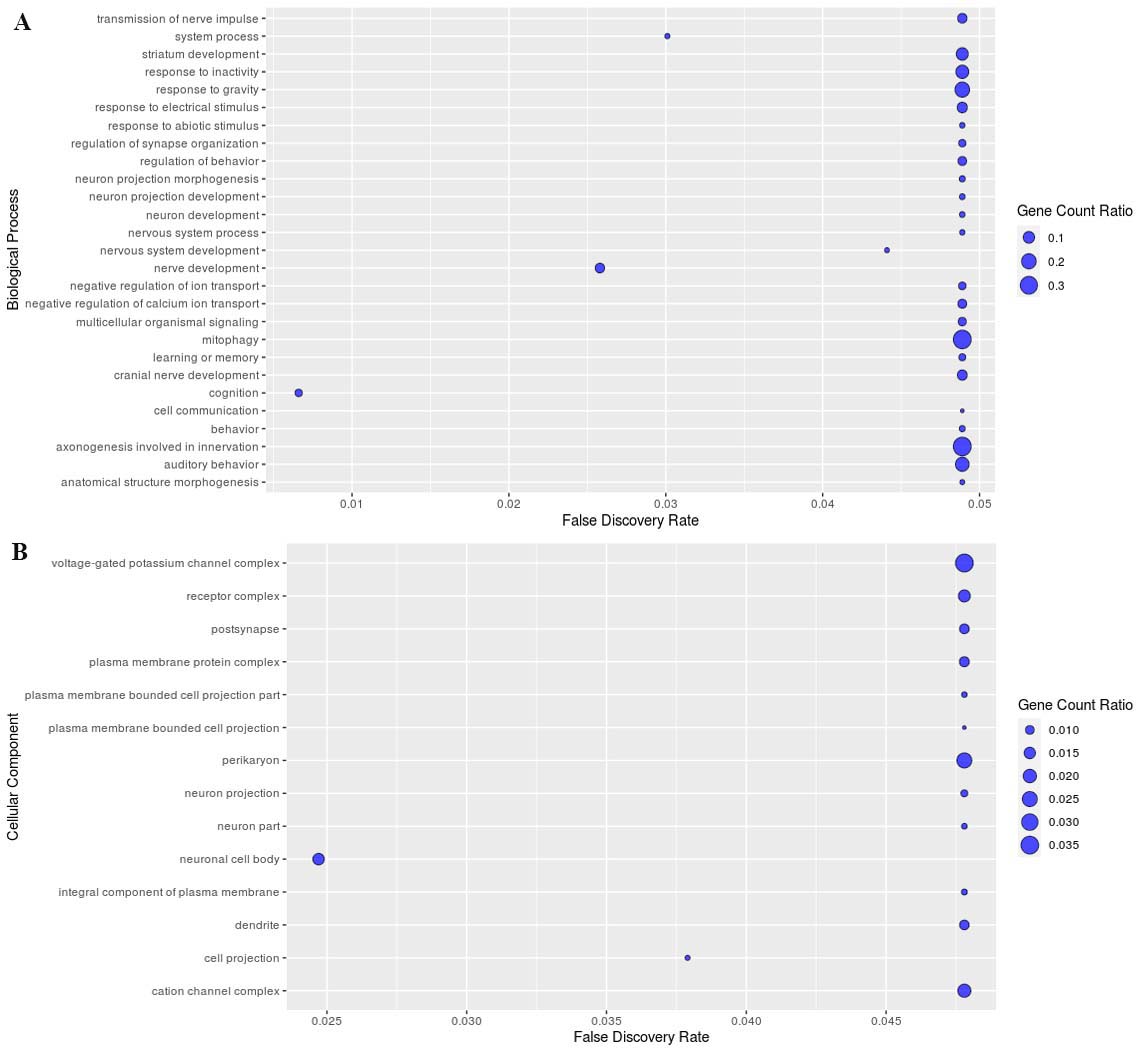

Supplement: Supplementary file 2 — Supplementary Information 2. [file 41598_2021_89798_MOESM2_ESM.jpg]
