# Supplementary material for: Genomic selection signatures in autism spectrum disorder identifies cognitive genomic tradeoff and its relevance in paradoxical phenotypes of deficits versus potentialities
Source: Sci Rep. 2021 May 13;11:10245. doi: 10.1038/s41598-021-89798-w (PMC8119484; doi:10.1038/s41598-021-89798-w)
Supplement: Supplementary file 4 — Supplementary Information 4. [file 41598_2021_89798_MOESM4_ESM.docx]

**Genomic selection signatures in Autism identifies cognitive genomic tradeoff and its relevance in paradoxical phenotypes of Autism**

Anil Prakash^1,2^ and Moinak Banerjee^*^_’_^1^

^1^Neurobiology and Genetics Division, Rajiv Gandhi Center for Biotechnology, Thiruvananthapuram, Kerala, 695014, INDIA

^2^Department of Biotechnology, University of Kerala, Kariavattom, Thiruvananthapuram, Kerala, India

**SUPPLEMENTARY FILES**

**SUPPLEMENTARY TABLES**

**Supplementary Table S1:** Autism risk SNPs selected from SFARI database

**Supplementary Table S2:** Identifying selection using Hierarchical Boosting selection method in Autism risk SNPs, Positive and Negative controls SNPs in three major ethnic population CEU, CHD and YRI in Phase I data using 1000 genome selection browser

**Supplementary Table S3:** Individual test for selection in Autism risk SNPs, Positive and Negative controls SNPs in three major ethnic population CEU, CHD and YRI in Phase I data using 1000 genome selection browser**.** Fixation index (Wright’s FST), Tajima’s D, difference of derived allele frequency(DDAF), cross-population extended haplotype homozygosity (XPEHH), cross-population composite likelihood ratio (XPCLR) and integrated haplotype score (iHS), (window size varies according to the test)

**Supplementary Table S4:** Individual test for selection in Autism risk SNPs, Positive and Negative controls SNPs in three major ethnic population CEU, CHD and YRI in Phase III data using PopHuman selection browser**.** Fixation index (Wright’s FST), cross-population extended haplotype homozygosity (XPEHH), and integrated haplotype score (iHS) (window size varies according to the test)

**Supplementary Table S5:** Positively selected Autism risk SNPs based Bayesian conjugate beta-binomial analysis

**Supplementary Table S6:** Functional prediction of Autism risk SNPs based on RegulomeDB rank, SIFT score and PolyPhen score

**Supplementary Table S7:** Functional prediction of expression profile based on eQTL data retrieved from GTEx portal

**Supplementary Table S8:** Gene Ontology enrichment analysis to understand the biological processes, cellular components and pathways using STRING analysis

**Supplementary Table S9:** Ancient genome analysis using Chimpanzee, Gorilla, Orangutan, Neanderthals, Denisovans and early Modern humans sampled from 4500 - 45,000 YBP to recent modern humans.

**SUPPLEMENTARY FIGURES**

**Supplementary Figure S1:** Chromosome and locus specific presentation of number of positive selection tests in Autism risk loci (Blue diamonds), Negative control loci (Green triangles) and Positive control loci (Red circle). Threshold of three positive test is represented by horizontal line.

**Supplementary Figure S2:** Gene ontology enrichment plots for eQTL genes harboring the positively selected SNPs show involvement of (A) biological and (B) cellular processes with their FDR cut off and gene count ratio.
